# A useful EGFR-TK ligand for tumor diagnosis with SPECT: development of radioiodinated 6-(3-morpholinopropoxy)-7-ethoxy-4-(3′-iodophenoxy)quinazoline

**DOI:** 10.1007/s12149-013-0703-y

**Published:** 2013-03-15

**Authors:** Masahiko Hirata, Yasukazu Kanai, Sadahiro Naka, Mitsuyoshi Yoshimoto, Shinya Kagawa, Keiji Matsumuro, Hideyuki Katsuma, Hiroshi Yamaguchi, Yasuhiro Magata, Yoshiro Ohmomo

**Affiliations:** 1 Osaka University of Pharmaceutical Sciences, 4-20-1 Nasahara, Osaka Takatsuki, 569-1094 Japan; 2 Department of Molecular Imaging in Medicine, Osaka University Graduate School of Medicine, Yamadaoka 2-2, Osaka Suita, 565-0871 Japan; 3 Functional Imaging Division, National Cancer Center Hospital East, 6-5-1 Kashiwanoha, Chiba Kashiwa, 277-8577 Japan; 4 Research Institute, Shiga Medical Center 5-4-30 Moriyama, Shiga Moriyama, 524-8524 Japan; 5 Department of Molecular Imaging, Applied Medical Photonics Laboratory, Medical Photonics Research Center, Hamamatsu University School of Medicine, 1-20-1 Handayama, Higashi-ku, Hamamatsu, 431-3192 Japan

**Keywords:** EGF, Radiopharmaceutical, SPECT, Tyrosine kinase, Quinazoline, Radioiodine

## Abstract

**Objective:**

Epidermal growth factor receptor tyrosine kinase (EGFR-TK) represents an attractive target for tumor diagnosis agents. Previously, radioiodinated 4-(3-iodophenoxy)-6,7-diethoxyquinazoline (PHY) was reported to possess good characteristics as a tumor imaging agent. We have explored the feasibility of developing tumor diagnosis ligands superior to radioiodinated PHY.

**Methods:**

New phenoxyquinazoline derivatives were designed with various side chains introduced to the 6th position of PHY. The IC_50_ values of the new derivatives to interrupt EGFR-TK phosphorylation were evaluated and compared to well-known EGFR-TK inhibitors. Tumor uptake studies of the new ^125^I-labeled derivatives were conducted with A431 tumor-bearing mice. Selectivity and binding characteristics were analyzed by in vitro blocking studies and a binding assay. Furthermore, SPECT/CT scans were performed using A431 tumor-bearing mice.

**Results:**

Six quinazoline derivatives were designed and synthesized, and among these, **6a–d** were found to have relatively high EGFR-TK inhibitory potency. In tumor uptake studies, [^125^I]**6a (**[^125^I]PYK) was found to have the highest tumor uptake and longest retention in tumors. In contrast, [^125^I]PYK was rapidly cleared from peripheral tissues, resulting in a high tumor-to-tissue ratio 24 h after injection. Moreover, the EGFR-TK selectivity of [^125^I]PYK was confirmed by pretreatment experiments with specific EGFR-TK inhibitors. Furthermore, [^125^I]PYK provided clear SPECT images of tumors.

**Conclusions:**

Radioiodinated PYK, one of the newly synthesized quinazoline derivatives, was found to be a desirable ligand for EGFR-TK SPECT imaging. [^125^I]PYK showed high tumor accumulation and selective EGFR-TK binding and also succeeded in delivering high contrast imaging of tumors. These favorable characteristics of [^125^I]PYK suggest that the ^123^I-labeled counterpart, [^123^I]PYK, would have great potential for diagnostic SPECT tumor imaging.

## Introduction

Epidermal growth factor receptor (EGFR) plays a key role in signal transduction pathways that regulate fundamentally important cellular functions. EGFR consists of an outer membrane EGF binding site and an inner membrane tyrosine kinase domain (EGFR-TK). EGFR-TK phosphorylation is stimulated by epidermal growth factor (EGF) or transforming growth factor α (TGFα) binding to the extracellular ligand-binding domain of EGFR and subsequent receptor dimerization. The intracellular tyrosine residues then undergo phosphorylation, which is followed by second messenger signaling and subsequent downstream events that lead to cellular proliferation, differentiation, motility, adhesion, and apoptosis [1, 2]. EGFR is often overexpressed in cancers [3–6], thus inhibition of EGFR-TK as a target for cancer chemotherapy has proven to be effective in both preclinical and clinical settings.

EGFR-TK represents an attractive target for the development of new antitumor agents [7, 8]. Among the EGFR-TK inhibitors used or tested clinically as antitumor agents are the quinazoline derivatives ZD1839 (Astra-Zeneca) [9, 10] and OSI774 (Pfizer) [10, 11]. One of the most potent compounds in the EGFR-TK inhibitor series is the quinazoline derivative PD153035 [12, 13], which exhibits well-defined selectivity and tremendously high affinity for EGFR-TK (*K*_*i*_: 5 pM) coupled with remarkable cytotoxicity against several types of tumors in vitro such as the A431 epidermoid carcinoma cell line (IC_50_: 29 pM). In an effort to develop new cancer diagnostic agents, several PET and SPECT ligands have been synthesized and evaluated for imaging of EGFR-TK. The feasibility of targeting EGFR for tumor imaging has been experimentally demonstrated using ^18^F or ^11^C-labeled EGFR-TK inhibitors [14–20]; however, these labeled compounds were also observed to produce high radioactivity in the blood and peripheral tissues. Therefore, tumor images with these radioligands were affected by the radioactivity in peripheral tissues a short time after injection.

**Fig. 1:**
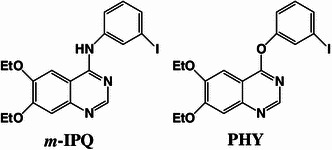
Structures of *m*-IPQ and PHY

We have previously synthesized and evaluated several radioiodinated quinazoline derivatives as new EGFR-TK imaging ligands (*m*-IPQ: 4-(3-Iodoanilino)-6,7-diethoxyquinazoline (IC_50_ value; 50.5 ± 3.5 nM), PHY: 4-(3-Iodophenoxy)-6,7-diethoxyquinazoline (IC_50_ value; 49.0 ± 7.2 nM), Fig. 1) [21, 22]. In vivo stability of [^125^I]PHY improved compared to [^125^I]*m*-IPQ. However, [^125^I]PHY showed low values of tumor-to-blood ratio (0.94–1.50) and tumor-to-muscle ratio (1.02–1.95). Accordingly, the properties of [^125^I]PHY was not good enough for in vivo molecular imaging probe. Therefore, we have been investigated the new EGFR-TK imaging probes superior to PHY in tumor accumulation and retention by the ligand structure modification.

In this paper, several new quinazoline derivatives were designed on the basis of essential requirements for radiophamaceuticals and structure–activity relationships. New ^125^I-labeled derivatives with various side chains introduced into the 6th position of PHY were synthesized, and biological studies with these compounds were carried out including analysis of SPECT/CT images. From these results, the new radioiodinated derivatives were evaluated for their use in SPECT tumor imaging.

## Materials and methods

[^125^I]NaI and [^32^P]ATP were purchased from Tokyo Biomedical (Tokyo, Japan) and Muromachi Yakuhin (Tokyo, Japan), respectively. All other chemicals were of reagent grade and were purchased from Sigma-Aldrich Japan (Tokyo, Japan), Nacalai Tesque (Kyoto, Japan), Wako Pure Chemical Industry (Osaka, Japan) or Tokyo Chemical Industry (Tokyo, Japan). 4-(3-Iodophenoxy)-6,7-diethoxyquinazoline (**1**, PHY) was synthesized according to our previous paper [22]. 4-(3-chloropropyl) morpholine and 1-(3-chloropropyl)-4-methylpiperazine were synthesized by the method of Adams et al. [23]. The HPLC system employed included a M-600 pump, a Lambda-Max 481 ultraviolet detector (Waters, MA), a 170 NaI radioactivity detector (Beckman, CA), and a _5_C_18_-AR column (10 × 250 mm, Nacalai Tesque, Kyoto, Japan). Male ddY mice and balb/c nude mice were obtained from Japan SLC Inc. (Shizuoka, Japan). Animals were housed with ad libitum access to food and drinking water for at least one week before initiating experiments. Animals were housed and experiments were performed according to guidelines stipulated by the Osaka University of Pharmaceutical Sciences Animal Care and Use Committee.

### 6-Hydroxy-7-ethoxyquinazolin-4(3H)-one (**2**)

A mixture of 6,7-diethoxyquinazolin-4(3H)-one (9.2 g, 39.4 mmol) and l-methionine (7.05 g, 47.3 mmol) was dissolved in methanesulfonic acid (60 mL) and heated to 100 °C for 4 h. The mixture was cooled to room temperature, poured onto a mixture (200 mL) of ice/water, and neutralized by the addition of aqueous sodium hydroxide solution (40 %) resulting in precipitation of a white deposit. This product was collected by filtration using a sintered glass funnel, washed with water, and was used without further purification.

### 6-Acetoxy-7-ethoxyquinazoline 4(3H)-one (**3**)

A stirred suspension of 6-hydroxy-7-methoxyquinazolin-4(*3H*)-one (**2**, 7.3 g, 35.4 mmol) in acetic anhydride (40 mL), pyridine (6.0 mL) and *N*,*N*-dimethyl-4-aminopyridine (100 mg, 0.819 mmol) was heated to 100 °C for 6 h. Crushed ice/water solution (150 mL) was added to the reaction mixture, and the resulting white precipitate was filtered and washed with water.

Yield; 61.8 %. mp 258–260 °C. ^1^H-NMR (DMSO); 8.07 (s, 1H, aromatics), 7.73 (s, 1H, aromatics), 7.24 (s, 1H, aromatics), 4.19 (q, *J* = 7.6 Hz, 2H, CH_3_C**H**_**2**_O), 2.30 (s, 3H, C**H**_**3**_CO), 1.33 (t, *J* = 7.6 Hz, 3H, C**H**_**3**_CH_2_O). MS: *m/z* 248; found 248.

### 4-Chloro-6-acetoxy-7-ethoxyquinazoline-6-yl acetate (**4**)

A stirred solution of 7-ethoxy-4-oxo-3,4-dihydroquinazolin-6-yl acetate (**3**, 2.0 g, 22.7 mmol) and *N*,*N*-diethylaniline (28.5 mL) in phosphoryl chloride (30 mL) was immersed in a preheated oil bath at 100 °C for 30 min. The reaction mixture was stirred for a further 30 min at 80 °C. The phosphoryl chloride was removed, and the crude material was triturated with abs. toluene (3 × 30 mL). Crushed ice/water was added (200 mL) to the reaction mixture, and the light brown precipitate was filtered and washed with ice water (100 mL). The brownish precipitate was air-dried.

Yield; 89.6 %. mp 110–114 °C. ^1^H-NMR (CDCl_3_): 8.93 (s, 1H, aromatics), 7.88 (s, 1H, aromatics), 7.40 (s, 1H, aromatics), 4.25 (q, *J* = 7.6 Hz, 2H, CH_3_C**H**_**2**_O), 2.39 (s, 3H, C**H**_**3**_CO), 1.50 (t, *J* = 7.6 Hz, 3H, C**H**_**3**_CH_2_O). MS: *m/z* 266; found 266.

### 7-Ethoxy-6-hydroxy-4-(3′-iodophenoxy)quinazoline (**5**)

3-Iodophenol (6.0 g, 27.3 mmol) and KOH (6.0 g, 27.3 mmol) were heated at 50 °C, and then **4** (0.5 g, 2 mmol) was added slowly over 10 min. After cooling, the reaction mixture was dissolved in CHCl_3_ (50 mL), the solution was washed with 100 mL of water, and then the organic layer was dried with Na_2_SO_4_ and evaporated to give an oily residue that was purified by silica gel column chromatography (MeOH/CHCl_3_ = 1/10) to afford the desired compound.

Yield; 71.2 %. mp 214–216 °C. ^1^H-NMR (DMSO); 10.30 (s, 1H, O**H**), 8.48 (s, 1H, aromatics), 7.73 (t, 1H, aromatics), 7.68 (d, 1H, aromatics), 7.48 (s, 1H, aromatics), 7.30 (m, 3H, aromatics), 4.25 (q, *J* = 7.6 Hz, 2H, CH_3_C**H**_**2**_O) 1.44 (t, *J* = 7.6 Hz, 3H, C**H**_**3**_CH_2_O). HRMS: *m/z* 407.9971; found 407.9978.

### 6-(3-Morpholinopropoxy)-7-ethoxy-4-(3′-iodophenoxy)quinazoline (**6a**) [PYK]

A solution of **5** (0.60 g, 1.47 mmol) and potassium carbonate (1.0 g, 7.24 mmol) in DMF (15 mL) was stirred at 40 °C for 20 min, and then 4-(3-chloropropyl)morpholine was added. This mixture was stirred and heated at 90 °C for 3.5 h. The reaction mixture was poured onto a crushed ice/water mixture and extracted with ethyl acetate (3 × 50 mL). The organic layers were combined, extracted with brine (2 × 100 mL), dried over Na_2_SO_4_, and the solvent was removed. The crude material was recrystallized from methanol to afford the product.

Yield; 38.1 %. mp 135–137 °C. ^1^H-NMR (CDCl_3_); 8.54 (s, 1H, aromatics), 7.59 (t, 1H, aromatics), 7.56 (d, 1H, aromatics), 7.43 (s, 1H, aromatics), 7.18 (m, 3H, aromatics), 4.20 (t, *J* = 8.0 Hz, 2H, propyl), 4.17 (q, *J* = 7.6 Hz, 2H, CH_3_C**H**_**2**_O), 3.66 (t, *J* = 5.0 Hz, 4H, morpholino), 2.53 (t, *J* = 8.0 Hz, 2H, propyl), 2.43 (t, *J* = 5.0 Hz, 4H, morpholino), 2.06 (m, *J* = 8.0 Hz, 2H, propyl), 1.49 (t, *J* = 7.6 Hz, 3H, C**H**_**3**_CH_2_O). HRMS: *m/z* 535.0968; found 535.0966.

### 6-[3-(4-Methylpiperazinylpropoxy)]-7-ethoxy-4-(3′-iodophenoxy)quinazoline (**6b**)

**6b** was similarly obtained from 1-chloro-3-(1-methyl-4-piperazinyl)propane (**10)** as a starting material.

Yield; 53.6 %. mp 107–109 °C. ^1^H-NMR (CDCl_3_); 8.60 (s, 1H, aromatics), 7.67 (t, 1H, aromatics), 7.63 (d, 1H, aromatics), 7.50 (s, 1H, aromatics), 7.23 (m, 3H, aromatics), 4.27 (t, *J* = 6.0 Hz, 2H, propyl), 4.25 (q, *J* = 7.6 Hz, 2H, CH_3_C**H**_**2**_O), 2.60 (t, *J* = 6.0 Hz, 2H, propyl), 2.50 (broad s, 8H, piperazine), 2.28 (s, 3H, methyl), 2.12 (m, *J* = 8.0 Hz, 2H, propyl), 1.56 (t, *J* = 7.6 Hz, 3H, C**H**_**3**_CH_2_O). HRMS: *m/z* 548.1284; found 548.1279.

### 6-Isopropoxy-7-ethoxy-4-(3′-iodophenoxy)quinazoline (**6c**)

**6c** was similarly obtained with isopropyl chloride.

Yield; 45.3 %. mp 59–62 °C. ^1^H-NMR (CDCl_3_); 8.53 (s, 1H, aromatics), 7.58 (t, 1H, aromatics), 7.56 (d, 1H, aromatics), 7.46 (s, 1H, aromatics), 7.16 (m, 3H, aromatics), 4.67 (m, 1H, isopropyl), 4.19 (q, *J* = 7.6 Hz, 2H, CH_3_C**H**_**2**_O), 1.56 (t, *J* = 7.6 Hz, 3H, C**H**_**3**_CH_2_O) 1.41 (d, 6H, isopropyl). HRMS: *m/z* 450.0440; found 450.0428.

### 6-(3-Dimethylaminopropoxy)-7-ethoxy-4-(3′-iodophenoxy) quinazoline (**6d**)

**6d** was similarly obtained with 3-dimethylaminopropyl chloride

Yield; 88.6 %. mp 64–67 °C. ^1^H-NMR (CDCl_3_); 8.54 (s, 1H, aromatics), 7.75 (t, 1H, aromatics), 7.69 (d, 1H, aromatics), 7.30 (s, 1H, aromatics), 7.18 (m, 3H, aromatics), 4.25 (q, *J* = 7.6 Hz, 2H, CH_3_C**H**_**2**_O), 4.19 (t, *J* = 7.0 Hz, 2H, propyl), 2.39 (t, *J* = 7.0 Hz, 2H, propyl), 2.51 (s, 6H, dimethyl), 1.94 (m, *J* = 7.0 Hz, 2H, propyl), 1.43 (t, *J* = 7.6 Hz, 3H, C**H**_**3**_CH_2_O). HRMS; *m/z* 493.0862; found 493.0854.

### 6-(3-Phenylpropoxy)-7-ethoxy-4-(3′-iodophenoxy)quinazoline (**6e**)

**6e** was similarly obtained with 3-phenylpropyl chloride.

Yield; 57.4 %. mp 108–109 °C. ^1^H-NMR (CDCl_3_); 8.61 (s, 1H, aromatics), 7.67 (t, 1H, aromatics), 7.63 (d, 1H, aromatics), 7.45 (s, 1H, aromatics), 7.34–7.15 (m, 8H, aromatics), 4.28 (q, *J* = 7.6 Hz, 2H, CH_3_C**H**_**2**_O), 4.18 (t, *J* = 11.6 Hz, 2H, propyl), 2.89 (t, *J* = 11.6 Hz, 2H, propyl), 2,27 (m, *J* = 11.6 Hz, 2H, propyl), 1.43 (t, *J* = 7.6 Hz, 3H, C**H**_**3**_CH_2_O). HRMS; *m/z* 526.0753; found 526.0754.

### 6-Benzyloxy-7-ethoxy-4-(3′-iodophenoxy)quinazoline (**6f**)

**6f** was similarly obtained with benzyl chloride.

Yield; 73.7 %. mp 140–142 °C. ^1^H-NMR (CDCl_3_); 8.54 (s, 1H, aromatics), 7.58 (t, 1H, aromatics), 7.55 (d, 1H, aromatics), 7.46 (s, 1H, aromatics), 7.40 (d, 1H, aromatics), 7.38 (s, 1H, aromatics), 7.35–7.20 (m, 4H, aromatics), 7.18 (t, 1H, aromatics), 7.15 (t, 1H, aromatics), 4.21 (q, *J* = 7.6 Hz, 2H, CH_3_C**H**_**2**_O), 1.50 (t, *J* = 7.6 Hz, 3H, C**H**_**3**_CH_2_O). HRMS; *m/z* 498.0440; found 498.0437.

### 4-[(3′-Tributylstannylphenoxy]-7-ethoxyquinazoline 6-substituted derivatives (**7a–d**)

A mixture of phenoxyquinazoline derivative (**6a–d**, 0.28 mmol), bistributyltin (0.49 g, 0.84 mmol), and tetrakis(triphenylphosphine)palladium (0.02 g, 0.01 mmol) in anhydrous toluene (25 mL) was refluxed under argon for 18 h. After cooling, the reaction mixture was filtered through Celite. The filtrate was concentrated *in vacuo*, and the oily residue was purified by silica gel column chromatography (MeOH/CHCl_3_ = 1/15 to afford the desired compound (**7a–d**).

### 7-Ethoxy-6-(3-morpholinopropoxy)-4-(3-tributylstannylphenoxy)quinazoline (**7a**)

Yield; 35.8 %. ^1^H-NMR (CDCl_3_); 8.60 (s, 1H, aromatics), 7.60 (s, 1H, aromatics), 7.49–7.15 (m, 5H, aromatics), 4.27 (t, *J* = 8.0 Hz, 2H, propyl), 4.25 (q, *J* = 7.6 Hz, 2H, CH_3_C**H**_**2**_O), 3.75 (t, *J* = 5.0 Hz, 4H, morpholino), 2.63 (t, *J* = 8.0 Hz, 2H, propyl), 2.54 (t, *J* = 5.0 Hz, 4H, morpholino), 2.15 (m, *J* = 8.0 Hz, 2H, propyl), 1.55 (t, *J* = 7.6 Hz, 3H, C**H**_**3**_CH_2_O), 1.42–0.86 (m, 27H, Bu_3_). HRMS: *m/z* 699.3058; found 699.3054.

### 7-Ethoxy-6-[3-(4-methylpiperazinylpropoxy)]-4-(3-tributylstannylphenoxy)quinazoline (**7b**)

Yield; 66.8 %. ^1^H-NMR (CDCl_3_); 8.60 (s, 1H, aromatics), 7.57 (s, 1H, aromatics), 7.47–7.35 (m, 4H, aromatics), 7.17 (t, 1H, aromatics), 4.27 (t, *J* = 6.0 Hz, 2H, propyl), 4.25 (q, *J* = 7.6 Hz, 2H, CH_3_CH_2_O), 2.60 (t, *J* = 6.0 Hz, 2H, propyl), 2.65 (broad s, 8H, piperazino), 2.34 (s, 3H, methyl), 2.14 (m, *J* = 8.0 Hz, 2H, propyl), 1.56 (t, *J* = 7.6 Hz, 3H, CH_3_CH_2_O), 1.39–0.86 (m, 27H, Bu_3_). HRMS: *m/z* 712.3374; found 712.3368.

### 7-Ethoxy-6-isopropoxy-4-(3-tributylstannylphenoxy)quinazoline (**7c**)

Yield; 53.9 %. ^1^H-NMR (CDCl_3_); 8.60 (s, 1H, aromatics), 7.61 (t, 1H, aromatics), 7.46–7.12 (m, 5H, aromatics), 4.75 (m, 1H, isopropyl), 4.27 (q, *J* = 7.6 Hz, 2H, CH_3_C**H**_**2**_O), 1.60–0.86 (t, 30H, C**H**_**3**_CH_2_O and Bu_3_). HRMS: *m/z* 614.2530; found 614.2524.

### 6-(3-Dimethylaminopropoxy)-7-ethoxy-4-(3-tributylstannylphenoxy)quinazoline (**7d**)

Yield; 50.1 %. ^1^H-NMR (CDCl_3_); 8.60 (s, 1H, aromatics), 7.60 (s, 1H, aromatics), 7.46–7.16 (m, 5H, aromatics), 4.25 (q, *J* = 7.6 Hz, 2H, CH_3_C**H**_**2**_O), 4.22 (t, *J* = 7.0 Hz, 2H, propyl), 2.52 (t, *J* = 7.0 Hz, 2H, propyl), 2.28 (s, 6H, dimethyl), 2.11 (m, *J* = 7.0 Hz 2H, propyl), 1.43 (t, *J* = 7.6 Hz, 3H, C**H**_**3**_CH_2_O), 1.37–0.86 (m, 27H, Bu_3_). HRMS: *m/z* 657.2952; found 657.2946.

## Radiolabeling

New radioiodinated quinazoline derivatives were prepared by an iododestannylation reaction under non-carrier-added conditions from the corresponding tributyltin precursor (**7a–d)** as outlined in scheme 2. Aqueous hydrogen peroxide (10 μL, 30 %) was added to a mixture of [^125^I]NaI (10 μL, 37.0 MBq, 74 TBq/mmol), 0.1 M HCl (25 μL) and the tributylstannyl precursor **7a–d** (0.01 mg in 10 μL ethanol) in a sealed vial. After stirring for 10 min at room temperature, the reaction mixture was quenched with aqueous sodium bisulfite (0.1 mg in 10 μL). The mixture was isolated by HPLC using 0.1 M citrate buffer/MeOH or 0.1 M NaH_2_PO_4_ buffer/MeOH as the eluent at a flow rate of 3.0 mL/min. The fraction corresponding to [^125^I]**6a–d** was collected, and the solvent was removed *in vacuo*. Retention times, radiochemical yields, and radiochemical purities of the tracers were measured by HPLC.

## Cell culture

A431 human carcinoma cell line was cultured in serum-supplemented Dulbecco’s modified Eagle’s medium (DMEM) containing 10 % heat-inactivated fetal bovine serum and penicillin/streptomycin mixed solution at 37 °C. The cells were split weekly in a 1:10 ratio using trypsin/EDTA. They were then transferred to 100 mm cell culture dishes and allowed to grow until reaching confluence (about 0.5 million cells). Subsequently, the cells were detached using either trypsin/EDTA (0.25 %, 1.0 mL) or by scraping with a cell scraper into DMEM when they reached sub confluence. NALM6 leukemia cell line [24] as a non-adherent cell was cultured in DMEM containing 10 % heat-inactivated fetal bovine serum and penicillin/streptomycin mixed solution at 37 °C. The cells were split weekly into a 1:10 ratio. They were then transferred to culture flask and allowed to grow until about 0.5 million cells were obtained.

## Measurement of inhibitory potency against EGFR-TK

The inhibitory potency of the new derivatives against EGFR-TK was measured using a Signa TECT^®^ Protein Kinase Assay System (Promega, WI) according to the protein tyrosine kinase assay protocol of the attached document. The EGF receptor used was an immunopurified receptor from human A431 cells (Promega, WI). The substrate used was based on a portion of biotinylated protein tyrosine kinase selective peptide. EGF receptors (1 μg of protein, 5 μL), sodium vanadate (2.5 mM, 2.5 μL), biotinylated protein tyrosine kinase selective peptide (2.5 mM, 2.5 μL) and the dissolved test compound in water including 1 % DMSO (desired concentration, 7.5 μL) were added to the PTK assay 5X buffer (5 μL), and the mixture was preincubated at 30 °C for 5 min. Next, [^32^P]ATP (0.5 mM ATP, 18.5 kBq, 2.5 μL) was added to the reaction mixture. The reaction was allowed to proceed for 10 min at 30 °C and then was stopped by the addition of guanidine hydrochloride solution (7.5 M, 13 μL). Each reaction mixture (12.5 μL) was spotted onto a biotin capture membrane. After all of the samples had been spotted, they were washed and rinsed as follows: 1 time for 30 s with 200 mL of 2 M NaCl, 3 times for 2 min each with 200 mL of 2 M NaCl, 4 times for 2 min each with 200 mL of 2 M NaCl in 1 % H_3_PO_4_, and 2 times for 30 s each with 100 mL of deionized water. The biotin capture membranes were dried on a piece of aluminum foil under a heat lamp for 5–10 min. The radioactivity trapped on the biotin capture membranes was counted with a liquid scintillation counter in 10 mL of scintillation fluid. The IC_50_ values were determined from sigmoid curves of the percent inhibition of [^32^P]ATP radioactivity.

## Tumor uptake studies of radioiodinated ligands using tumor-bearing mice

A suspension of 1 × 10^7^ A431 cells in 0.1 mL of medium was inoculated subcutaneously in male balb/c nude mice (4 weeks old, 20–25 g, *n* = 3). After approximately 2 weeks, when the solid tumor had grown to optimal size, the mice were used for tumor uptake studies. [^125^I]**6a–d** (37 kBq, 74 TBq/mmol) in 0.1 mL of saline was injected intravenously into tumor-bearing mice via the lateral tail vein. At 1 or 24 h after administration, the animals were sacrificed. Samples of tumor were excised and weighed. Radioactivity was measured using a well-type NaI(Tl) scintillation gamma counter. The results were expressed in terms of percentage of injected dose per gram of tumor.

## Biodistribution of the [^125^I]PYK using normal and tumor-bearing mice

[^125^I]PYK (37 kBq, 74 TBq/mmol) in 0.1 mL saline was injected intravenously into ddY male mice (5 weeks old, 25–30 g, *n* = 4) via the lateral tail vein. At the desired time (5 min—24 h) interval after administration, the animals were sacrificed by decapitation. Samples of blood and tissues of interest were excised and weighed. Radioactivity was measured using a well-type NaI(Tl) scintillation gamma counter. The results were expressed in terms of percentage of injected dose per gram of blood or tissue.

Next, biodistribution studies of [^125^I]PYK using A431 bearing balb/c nude mice (6 weeks old, 20–25 g, *n* = 4) were performed. The animals were euthanized 1, 6, 12 or 24 h after injection of the radioligands (37 kBq, 74 TBq/mmol in 0.1 mL saline), and the distributions were studied as described above.

## In vitro EGFR-TK selectivity of [^125^I]PYK

Crude P_2_ membrane fractions of A431 human carcinoma cell line (0.2 mg of protein, 10 μL) were incubated in HEPES (25 mM, pH 7.4, 880 μL) for 60 min at 25 °C with [^125^I]PYK (0.74 kBq, 10 μL, specific activity 74 TBq/mmol) and various inhibitors (1 μM, 100 μL): *m*-IPQ, PD153035, ZD1839, genistein, RG13022 (EGFR-TK), AG17 (platelet-derived growth factor receptor tyrosine kinase: PDGFR-TK), HNMPA (insulin-like growth factor receptor tyrosine kinase: IGFR-TK) or VEGFR inhibitor I. Assays were terminated by rapid vacuum filtration through Whatman GF/B glass fiber filters presoaked in 0.5 % polyethylenimine for at least 30 min at room temperature before use, and the assay filters were washed 8 times with 4 mL of ice-cold 0.1 M phosphate buffer (pH 7.4) before measuring the filter-bound radioactivity.

## Effect of inhibitor pretreatment on in vivo tumor uptake of [^125^I]PYK

For pretreatment experiments, tumor-bearing mice (6 weeks old, 20–25 g, *n* = 3) were given an intravenous injection of various inhibitors (1 mg/kg) 5 min before administration of [^125^I]PYK (37 kBq, 74 TBq/mmol). The animals were sacrificed 24 h after injection of the radioligands, and the ligand distribution was studied as described above. The differences between non-treatment and pretreatment data were examined by Student’s paired *t* tests.

## Binding assay of [^125^I]PYK

Assays were carried out as described in the previous section. Crude P_2_ membrane fractions (A431or NALM6, 0.1 mg of protein, 10 μL) and [^125^I]PYK (1–300 nM, 100 μL, 0.74 kBq) were used. Non-specific binding was determined in the presence of PD153035 (10 μM). Specific binding was defined as the difference between the total and non-specific binding. Scatchard plot analysis of the saturation binding data was used to determine the equilibrium dissociation constant (*K*_d_) and the maximal number of binding sites (*B*_*max*_).

## SPECT/CT imaging

The mice underwent SPECT/CT studies when A431 tumors had grown to optimal size (approximately 2 weeks after inoculation). ^125^I gamma camera imaging and image processing were performed using a small-animal imaging system with pinhole collimation (aperture diameter = 1 mm, focal length = 9 cm) and a 15- to 45 keV photo peak energy window (PET/SPECT/CT FX system, Gamma Medica Ideas). The A431 tumor-bearing mice were injected with approximately 14.8 MBq of [^125^I]PYK via the tail vein under isoflurane anesthesia. 24 h after injection, SPECT scans were performed at 64 projections over 360° (ROR = 4.5 cm, 60 s/projection). Reconstructed data from SPECT and CT were visualized and coregistered using AMIRA 3.1.

## Results

### Chemical synthesis

**Scheme 1:**
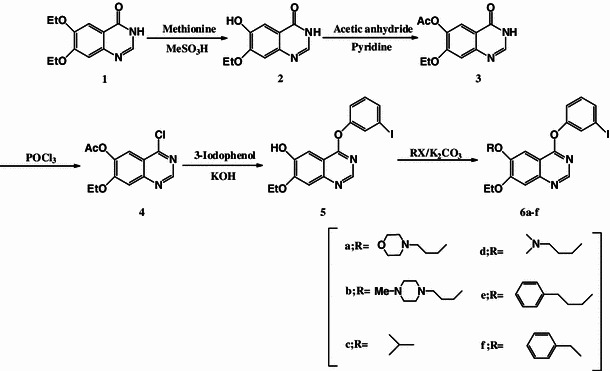
Synthesis of new quinazoline derivatives

4-(3-Iodophenoxy)-6,7-diethoxyquinazoline derivatives were designed and synthesized by the reaction scheme outlined in scheme 1. 4-Chloro-6,7-diethoxyquinazoline (**1**) was synthesized according to a modified procedure of Van Brocklin et al. [25]. Treatment with methanesulphonic acid in the presence of methionine dealkylated only the ethoxy group at the 6th position of **1**. The resulting free phenolic function of compound **2** was protected using acetic anhydride in the presence of pyridine to give compound **3**. Halogenation yielding the 4-chloro-compound **4** was performed with phosphoryl chloride according to the method of Rewcastle et al. [26]. Subsequently, reaction of **4** with 3-iodophenol in the presence of KOH yielded compound **5** following the method of Morley et al. [27]. Next, various side chains were introduced to the 6th position of the quinazoline (**6a–f**, 38.1–88.6 %).

### Inhibitory potency of new derivatives against EGFR-TK

The inhibitory potency of new phenoxyquinazoline derivatives against EGFR-TK in A431 cell membranes was measured as the inhibition of EGFR phosphorylation by [^32^P]ATP. The 50 % inhibition concentration (IC_50_) values are summarized in Table 1. Among the newly synthesized derivatives, **6a** and **6b** were found to have high potency (IC_50_; 12.0 ± 3.9, 16.5 ± 1.8 nM, respectively), slightly lower than that of PD153035 (IC_50_; 4.9 ± 1.29 nM). **6c** and **6d** were determined to possess comparable values to PHY (IC_50_; 41.5 ± 4.1, 113 ± 7.0 nM, respectively). On the other hand, **6e** and **6f** were found to have low inhibition of EGFR-TK (IC_50_; >500 nM, respectively). From these results, radiolabeling of **6a–d** was performed for further evaluation.

**Table 1:** IC_50_ of various inhibitors for EGFR-TK

Compounds	IC_50_ (nM)
**6a** (PYK)	12.0 ± 3.9
**6b**	16.5 ± 1.8
**6c**	113 ± 7.0
**6d**	41.5 ± 4.1
**6e**	>500
**6f**	>500
PD153035	4.9 ± 1.2
PHY	49.0 ± 7.2

Data represent triplicate determinations

### Radiolabeling

**Scheme 2:**
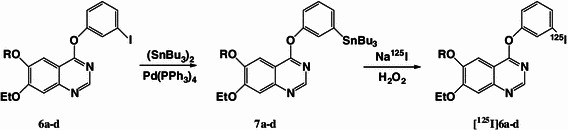
Radioiodination of quinazoline derivatives

New ^125^I-labeled phenoxyquinazoline derivatives were prepared by an iododestannylation reaction with the corresponding tributyltin precursor by the reaction outlined in scheme 2. ^125^I-labeling of the new ligands was achieved using hydrogen peroxide as an oxidant with [^125^I]NaI (specific activity 74 TBq/mmol) in 0.1 M HCl/ethanol solution at room temperature followed by HPLC purification. The HPLC retention times for [^125^I]**6a–d** were 10.6–23.4 min, and the radiochemical yields based on [^125^I]NaI ranged from 50.9 to 97.5 % (Table 2). ^125^I labeled **6a–c** were conveniently synthesized in high radiochemical yield (92.1–97.5 %). However, [^125^I]**6d** had a low radiochemical yield (50.9 %) compared with the other ligands presumably because **7d**, the precursor of [^125^I]**6d**, had poor solubility in EtOH/0.1 M HCl = 1/10. The radiochemical purities of these tracers were 99 %, and the specific activities were estimated to be approximately 74 TBq/mmol in theory since tin precursors were used and labeling was carried out under non-carrier-added conditions.

**Table 2:** HPLC analysis of ^125^I labeled quinazoline derivatives

Compounds	HPLC eluent*	Retention time (min)	Yield** (%)
[^125^I]**6a**	A/C = 45/55	15.5	97.5
[^125^I]**6b**	A/C = 45/55	18.8	92.1
[^125^I]**6c**	A/C = 50/50	23.4	92.4
[^125^I]**6d**	B/C = 10/90	12.7	50.9

* HPLC eluent, A: 0.01 M citric acid. B: 0.01 M NaH_2_PO_4_. C: methanol

** Yield: radiochemical yield

### Tumor uptake studies of the radioiodinated ligands

**Fig. 2:**
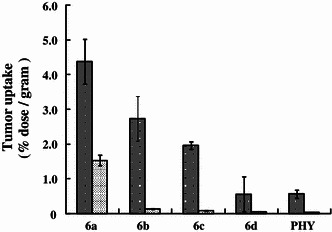
In vivo tumor uptake of ^125^I labeled phenoxyquinazoline derivatives in A431 tumor-bearing mice. Data represent mean ± SD, *n* = 3

In in vivo tumor uptake studies with A431 tumor-bearing mice (Fig. 2), the accumulation of [^125^I]**6a** (4.37 ± 0.65 % ID/g tissue), [^125^I]**6b** (1.96 ± 0.10 % ID/g tissue), and [^125^I]**6c** (2.73 ± 0.64 % ID/g tissue) in tumors 1 h post injection was higher than that of [^125^I]PHY (0.56 ± 0.12 % ID/g tissue), while uptake of [^125^I]**6d** (0.55 ± 0.12 % ID/g tissue) was similar to [^125^I]PHY. In addition, [^125^I]**6a** was found to have good retention in tumors (1.53 ± 0.15 % ID/g tissue 24 h after injection). On the other hand, [^125^I]**6b**, [^125^I]**6c**, and [^125^I]**6d** showed low retention in tumors 24 h after injection.

### Biodistribution of the [^125^I]PYK using normal and tumor-bearing mice

In vivo biodistribution studies of [^125^I]PYK were examined in male ddY mice at desirable time after intravenous administration and summarized in Table 3. [^125^I]PYK was transported well into various organs and disappeared slowly from the blood. In the liver highest uptake and rapid clearance were observed. The lungs, kidney, heart, pancreas, spleen, and brain uptakes were similar pattern to that of the liver. Furthermore, the increase in [^125^I]PYK uptake in the stomach and thyroid as deiodination parameters were not found. Therefore, it is suggested the good stability of [^125^I]PYK in in vivo.

**Table 3:** Biodistribution of [^125^I]PYK in normal mice

Organ	Time after injection						
	5 min	15 min	30 min	1 h	3 h	6 h	24 h
Blood*	0.98 ± 0.61	1.05 ± 0.08	0.80 ± 0.07	0.83 ± 0.16	0.50 ± 0.13	0.29 ± 0.03	0.08 ± 0.04
Pancreas	6.95 ± 0.49	5.52 ± 0.50	3.42 ± 0.33	2.47 ± 0.43	2.18 ± 0.92	1.53 ± 0.38	0.35 ± 0.05
Spleen	4.42 ± 0.95	4.23 ± 0.90	3.70 ± 1.24	2.44 ± 0.35	0.99 ± 0.56	1.08 ± 0.25	0.24 ± 0.02
Stomach	2.41 ± 0.59	3.03 ± 0.36	3.41 ± 0.37	4.33 ± 0.76	0.59 ± 0.23	0.73 ± 0.60	0.16 ± 0.04
Intestine	4.57 ± 0.63	5.57 ± 1.69	9.11 ± 0.57	15.84 ± 2.63	14.82 ± 7.80	8.53 ± 1.84	3.76 ± 1.38
Colon	1.89 ± 0.37	2.21 ± 0.48	2.33 ± 0.40	3.30 ± 0.10	21.14 ± 5.62	22.10 ± 7.54	15.91 ± 6.59
Liver	10.5 ± 0.92	13.9 ± 2.47	13.3 ± 1.83	12.3 ± 2.45	10.94 ± 7.67	4.70 ± 0.36	0.52 ± 0.10
Kidney	15.1 ± 1.38	15.6 ± 1.33	12.6 ± 2.36	7.94 ± 0.37	13.80 ± 9.25	3.58 ± 0.52	0.62 ± 0.10
Heart	3.47 ± 0.29	1.76 ± 0.89	1.43 ± 0.16	1.33 ± 0.23	0.64 ± 0.25	0.63 ± 0.21	0.22 ± 0.04
Lung	11.89 ± 2.86	6.85 ± 1.99	4.40 ± 1.60	3.42 ± 0.83	1.75 ± 0.18	1.77 ± 0.22	0.38 ± 0.09
Skin	0.68 ± 0.13	0.91 ± 0.13	1.02 ± 0.08	0.99 ± 0.06	0.47 ± 0.46	0.75 ± 0.46	0.17 ± 0.11
Brain	2.07 ± 0.07	3.15 ± 1.22	0.82 ± 0.33	0.47 ± 0.29	0.27 ± 0.05	0.11 ± 0.03	0.02 ± 0.06
Muscle	1.78 ± 0.13	0.97 ± 0.15	1.02 ± 0.53	0.65 ± 0.04	1.00 ± 1.27	0.25 ± 0.04	0.03 ± 0.01
Bone	1.13 ± 0.28	1.36 ± 0.39	0.60 ± 0.19	1.81 ± 2.40	0.25 ± 0.16	0.23 ± 0.02	0.04 ± 0.02
Thyroid**	0.05 ± 0.04	0.05 ± 0.03	0.01 ± 0.01	0.03 ± 0.01	0.05 ± 0.04	0.03 ± 0.02	0.01 ± 0.00

* Mean % injected dose ± SD per gram tissue of four mice

** Mean % injected dose ± SD per organ of four mice

**Fig. 3:**
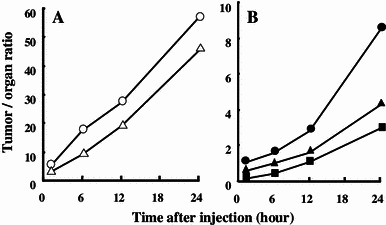
Tumor-to-organ ratios of [^125^I]PYK in A431 tumor-bearing mice **a**: tumor to blood (*open circle*), tumor to muscle (*open triangle*) **b**: tumor to lung (*closed circle*), tumor to kidney (*closed triangle*), tumor to liver (*closed square*)

Moreover, biodistribution studies of [^125^I]PYK were performed in A431 tumor-bearing mice with time points 1, 6, 12, and 24 h after intravenous administration. The results are summarized in Table 4. The accumulation of [^125^I]PYK in the tumor was high, and the tumor radioactivity level gradually decreased to 1.53 ± 0.15 % ID/g tissue from 1 to 24 h after imaging agent injection. In contrast with the high tumor uptake and retention of [^125^I]PYK, accumulation in the blood and muscle was low, which resulted in good tumor/blood (57.0, Fig. 3a) and tumor/muscle (45.5, Fig. 3a) ratios 24 h after injection. [^125^I]PYK also was found to have a relatively high uptake into the lungs, liver, and kidneys 1 h after injection; however, clearance of [^125^I]PYK from these organs was more rapid than from tumor tissue. In particular, the radioactivity accumulated in the liver 24 h after injection was approximately 1/50 of the radioactivity 1 h after injection. Thus, tumor-to-organ ratios for [^125^I]PYK 24 h after injection had desirable values (Fig. 3b: tumor to lung: 8.5, tumor to kidney: 4.3, tumor to liver: 3.0) for tumor diagnostic imaging.

**Table 4:** Biodistribution of [^125^I] PYK in A-431 tumor-bearing mice

Organ	Time after injection			
	1 h	6 h	12 h	24 h
Tumor*	4.37 ± 0.65	3.59 ± 0.73	1.72 ± 0.42	1.53 ± 0.15
Blood	0.80 ± 0.14	0.20 ± 0.04	0.06 ± 0.01	0.03 ± 0.00
Pancreas	2.80 ± 0.19	1.59 ± 0.25	0.41 ± 0.06	0.14 ± 0.02
Spleen	2.14 ± 0.04	1.02 ± 0.18	0.21 ± 0.01	0.07 ± 0.03
Stomach	6.10 ± 2.08	1.23 ± 0.60	0.69 ± 0.51	0.38 ± 0.19
Liver	27.0 ± 1.29	7.89 ± 1.45	1.55 ± 0.32	0.51 ± 0.35
Kidney	7.40 ± 0.66	3.53 ± 0.72	1.06 ± 0.14	0.36 ± 0.04
Heart	1.19 ± 0.17	0.50 ± 0.04	0.14 ± 0.02	0.05 ± 0.01
Lung	4.14 ± 0.48	2.29 ± 0.69	0.61 ± 0.18	0.18 ± 0.07
Brain	0.88 ± 0.07	0.17 ± 0.03	0.04 ± 0.01	0.02 ± 0.00
Muscle	1.34 ± 0.21	0.39 ± 0.02	0.09 ± 0.02	0.03 ± 0.01

* Mean % injected dose ± SD per gram of four mice

### In vitro EGFR-TK selectivity of [^125^I]PYK

**Fig. 4:**
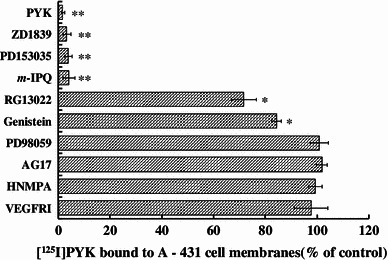
Blocking effect of various inhibitors on [^125^I]PYK binding to EGFR-TK. Data represent mean ± SD, *n* = 3 *p < 0.01, **p < 0.001

An in vitro pharmacological blocking study was performed to examine the degree of specific binding of [^125^I] PYK using A431 tumor cell membranes. As shown in Fig. 4, a significant reduction of [^125^I]PYK binding to EGFR-TK was found after pretreatment with EGFR-TK inhibitors such as *m*-IPQ, PD153035, and ZD1839. With genistein or RG13022 pretreatment, [^125^I]PYK binding was slightly decreased. The reduced radioactivity measured after treatment with these inhibitors correlated with the avidity of EGFR-TK. On the other hand, non-EGFR-TK inhibitors, such as AG17 (PDGFR-TK), HNMPA (IGFR-TK) and VEGFR inhibitor I (vascular endothelial growth factor tyrosine kinase), failed to block [^125^I]PYK binding to EGFR-TK. The difference between non-treatment and pretreatment data was examined by Student’s paired *t* test.

### Effect of pretreatment with various inhibitors on in vivo [^125^I]**6a** ([^125^I]PYK) uptake into tumors

**Fig. 5:**
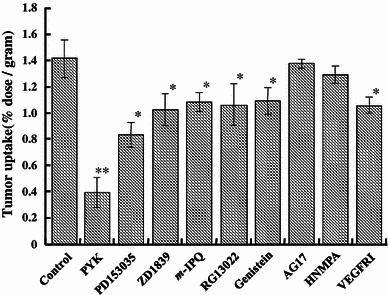
Effect of pretreatment with various inhibitors on in vivo tumor uptake of [^125^I]PYK. Data represent mean ± SD, *n* = 3 *p < 0.01, **p < 0.001

As shown in Fig. 5, a significant decrease of [^125^I]PYK uptake into tumors was found after pretreatment with EGFR-TK inhibitors. On the other hand, pretreatment with AG17 (PDGFR-TK) and HNMPA (IGFR-TK) had no effect on the tumor uptake of [^125^I]PYK; however, VEGFR inhibitor I was found to decrease its tumor uptake.

### Binding assay of [^125^I]PYK

**Fig. 6:**
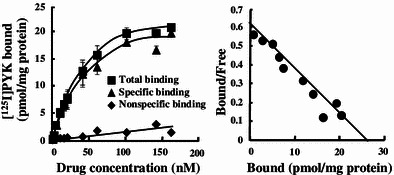
Binding assay of [^125^I]PYK to EGFR-TK in A431 cell membranes. **a** Binding curves of [^125^I]PYK; total binding (*closed circle*), specific binding (*closed triangle*), non-specific binding (*closed square*). **b** Scatchard plot of the binding. Data represent mean ± SD, *n* = 5

The crude P_2_ membrane fraction from A431 or NAlM6 cells were used in further binding experiments for pharmacological characterization. The saturation binding of [^125^I]PYK was analyzed using Scatchard analysis (Fig. 6), and the resulting linear Scatchard plot indicated that [^125^I]PYK bound to a single population of binding site with high affinity. Kinetic parameters from the analysis were *B*_max_ = 27.0 ± 3.43 pmol/mg protein and *K*_*d*_ = 51.3 ± 11.1 nM. While, in the NALM6 as a negative-EGFR leukemia cell line [24], [^125^I]PYK binding to cell membranes was undetectable.

### SPECT/CT imaging

**Fig. 7:**
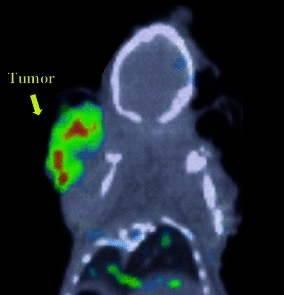
SPECT/CT image of [^125^I]PYK in A431 bearing mice 24 h after injection

Tumor images using [^125^I]PYK with animal-SPECT are shown in Fig. 7. [^125^I]PYK-SPECT images of tumors were very clear without interference from radioactivity in peripheral organs.

## Discussion

EGFR-TK activation is involved in the proliferation, invasion, metastasis, angiogenesis, and suppression of apoptosis of cancer cells [1, 2]. Therefore, EGFR-TK is an attractive molecular target for tumor diagnosis. Several radiolabeled quinazoline derivatives that have high inhibitory potency against EGFR-TK have been studied for diagnostic tumor imaging. Among these quinazoline EGFR-TK inhibitors, PD153035 has a very high inhibitory potency. The Parke-Davis Institute group and Tsou et al. have reported various structure–activity relationships of quinazoline derivatives [26, 28–33]. From their results, it was thought that various modifications of the quinazoline structure to provide a radiopharmaceutical would be possible. Thus, PD 153035 was selected as a lead compound for development of a new EGFR-TK diagnostic agent. We have previously reported radioiodinated 4-(3-iodophenoxy)-6,7-diethoxyquinazoline (PHY) as a phenoxyquinazoline derivative that has good characteristics for an EGFR-TK imaging ligand. In this study, our aim was to develop a radiopharmaceutical superior to radioiodinated PHY for tumor diagnoses that provided high tumor accumulation and selective interaction with the target molecule. On the basis of previous work in the development of various radiopharmaceuticals, several new phenoxyquinazoline derivatives with various side chains introduced on to the 6th position of the quinazoline were designed.

Six phenoxyquinazoline derivatives were easily synthesized from the key compound **5** by reactions outlined in Scheme 1. Inhibitory potencies of these derivatives for EGFR-TK were measured (Table 1). The IC_50_ values were measured for the new inhibitors as well as for PD153035 and traditional EGFR-TK inhibitors as standards, and the new inhibitors were compared with these standard compounds to evaluate their inhibitory potency. The four quinazoline derivatives **6a–d** were found to possess high inhibitory potency, while **6e** and **6f** displayed low inhibitory potency. In order to have high inhibitory potency against EGFR-TK, it is necessary that an inhibitor bind strongly to the ATP binding site. Thus, introduction of a hydrophilic side chain to the 6th position of the quinazoline providing a positive charge interaction was effective, particularly for hydrophilic side chains linked by a three carbon chain. These results were in agreement with previous structure–activity relationships [26, 28–33].

In initial screenings to assess the relative potential of the new radiolabeled quinazoline derivatives to act as diagnostic agents for tumor imaging by in vivo tumor uptake studies in A431 tumor-bearing mice (Fig. 2), [^125^I]**6a–c** showed higher accumulation in tumors than [^125^I]PHY. Accordingly, the goal of increasing the tumor accumulation of an imaging agent was achieved. In particular, [^125^I]**6a** showed the highest tumor accumulation, approximately 8 folds higher than [^125^I]PHY 1 h post injection. In addition, [^125^I]**6a** had good retention in tumors, whereas [^125^I]**6b–d** showed lower retention in tumors 24 h after injection. From these results, [^125^I]**6a** (i.e., PYK) showed good potential for EGFR-TK imaging, and therefore, further evaluation with this compound was performed.

In a biodistribution study (Tables 3, 4), accumulation of [^125^I]PYK in tumors was high, with gradual decrease in the tumor radioactivity level. In contrast with the high tumor uptake and retention of [^125^I]PYK, accumulation in peripheral organs was low, which resulted in good tumor to organ radioactivity ratios 24 h after injection. The clearance of [^125^I]PYK from these peripheral organs was a desirable attribute for a radiopharmaceutical. The tumor uptake of various radiolabeled quinazoline derivatives as PET ligands has previously been reported. These ligands were observed to have relatively high tumor uptake 1 h post injection [14–16]; however, they were also found to induce high radioactivity in peripheral tissues 1 h post injection. Therefore, the tumor images of these previous quinazoline derivatives were attenuated by the radioactivity of peripheral tissues a short time after injection. Compared with other radiolabeled quinazoline EGFR-TK ligands, the accumulation of [^125^I]PYK in tumors 1 h post injection (4.37 ± 0.65 % ID/g tissue) was much greater. In addition, radioiodine is possible to trace for a longer time than positrons. Thus, [^125^I]PYK is expected to give high contrast images of tumors for a long time post injection since [^125^I]PYK has a high tumor-to-organ ratio 24 h after injection (Fig. 3).

Selective interaction of [^125^I]PYK with EGFR-TK on A431 tumor cell membranes was confirmed by pretreatment experiments with various EGFR-TK and other tyrosine kinase inhibitors. As shown in Fig. 4, significant decreases in [^125^I]PYK binding were found after pretreatment with selective EGFR-TK inhibitors. In addition, the reduced rate of [^125^I]PYK binding correlated with the inhibitory potency of these competitive EGFR-TK inhibitors. Alternatively, non-EGFR-TK inhibitors failed to block [^125^I]PYK binding. In in vitro pretreatment study, the selectivity of [^125^I]PYK to EGFR-TK was very clear and there was no effect on [^125^I]PYK binding by the pretreatment of non-EGFR-TK. On the other hand, in in vivo, [^125^I]PYK tumor accumulation was found decreases by the EGFR-TK inhibitors pretreatment and a slight decrease by VEGFR inhibitor I pretreatment (Fig. 5). The reason about slight reduction of [^125^I]PYK tumor accumulation by VEGFR inhibitor I was not clear, but it is known that the VEGFR inhibition causes various pharmacologic interactions such as decrease in tumor blood vessel permeability, vasoconstriction on tumor blood vessel [34, 35]. Accordingly, it was considered that this phenomenon might be caused by pharmacologic interactions on tumor blood vessel with VEGFR inhibitor I pretreatment.

In the binding assay of [^125^I]PYK using A431 tumor cell membranes (Fig. 6), linear Scatchard plots indicated that [^125^I]PYK bound to a single population of binding sites with high affinity (*K*_d_ value; 51.3 ± 11.1 nM; Hill’s constant value; 1.02). On the other hand, in the NALM6 tumor cell as a negative control, the [^125^I]PYK binding to EGFR-TK in tumor cell membranes was undetectable. Quinazoline derivatives have been reported to bind to the ATP binding domain of EGFR-TK [12, 26, 36, 37]. Hence, [^125^I]PYK presumably binds to the same ATP binding site analogous to other quinazoline derivatives.

These in vivo and in vitro data suggest that [^125^I]PYK has desirable characteristics for EGFR-TK SPECT imaging. Accordingly, our design of new EGFR-TK ligands for tumor diagnosis was appropriate. In fact, the [^125^I]PYK-SPECT imaging of tumors was very clear without radioactivity in the lungs as a ROI (Fig. 7). Thus, [^125^I]PYK might be useful as a diagnostic SPECT imaging agent for lung cancer.

Radioiodinated PYK showed high contrast imaging of tumors with EGFR-TK selectivity. Therefore, radioiodinated PYK-SPECT is expected to allow visualization of primary and metastatic tumor lesions that express active mutant EGFR-TK. Currently, we are exploring the feasibility of using radioiodinated PYK as a predictor for NSCLC therapy with gefitinib, and results of this study will be reported soon.

## Conclusion

The iodinated quinazoline derivatives (**6a–d**) were found to have relatively high inhibitory potency against EGFR-TK. [^125^I]**6a–d** were conveniently synthesized from tributylstannyl precursors by an iododestannylation reaction using [^125^I]NaI and hydrogen peroxide. From tumor uptake studies, the morpholinopropyl phenoxyquinazoline derivative (**6a**, [^125^I]PYK) was observed to have the highest in vivo tumor uptake. Furthermore, the tumor uptake of [^125^I]PYK was prolonged up to 24 h. [^125^I]PYK displayed selective binding to EGFR-TK. Tumor images using [^125^I]PYK-SPECT were very clear and could be useful for visualizing lung cancers. Thus, radioiodinated PYK appears to be a very useful tumor diagnostic radiopharmaceutical.
